# Impact of Surgical Timing on Functional Outcomes after Anterior Cruciate Ligament Reconstruction

**DOI:** 10.3390/jcm13102994

**Published:** 2024-05-20

**Authors:** Tatsuhiro Kawashima, Hirotaka Mutsuzaki, Arata Watanabe, Kotaro Ikeda, Yuki Yamanashi, Tomonori Kinugasa

**Affiliations:** 1 Department of Rehabilitation, Ichihara Hospital, Tsukuba 300-3295, Japan; kawatatsu1208@yahoo.co.jp; 2 Center for Medical Science, Ibaraki Prefectural University of Health Sciences, Ami 300-0394, Japan; 3 Department of Orthopedic Surgery, Ibaraki Prefectural University of Health Sciences Hospital, Ami 300-0331, Japan; 4 Department of Orthopedic Surgery, Ichihara Hospital, Tsukuba 300-3295, Japan; 5 Department of Orthopedic Surgery, University of Tsukuba, Tsukuba 305-8575, Japan; 6 Department of Orthopedic Surgery, Aichi Medical University, Nagakute 480-1195, Japan

**Keywords:** muscle strength, performance, return to sport

## Abstract

**Objectives**: Although acute anterior cruciate ligament reconstruction (ACLR) is often avoided because of postoperative joint stiffness, delayed ACLR can lead to a longer recovery time and can have a negative impact on physical function due to detraining. This study aimed to determine the effects of acute ACLR on postoperative outcomes, including muscle strength, performance, and return to sports. **Methods**: A total of 110 patients who underwent anatomical ACLR using hamstring autografts were included in this study and were divided into three groups: acute (ACLR performed within 2 weeks after ACL injury), 2–6 weeks (ACLR performed between 2 and 6 weeks after injury), and 6–12 weeks (ACLR performed between 6 and 12 weeks after injury). Several parameters were evaluated, including range of motion, knee joint stability, isokinetic knee strength, performance, and return to sports. **Results**: No significant differences were found in the range of motion or knee joint stability between the groups. The acute group exhibited significantly greater quadriceps strength at 3 months postoperatively than the other groups (*p* < 0.05). The single-leg hop test showed that 66.7%, 38.7%, and 33.3% of the patients in the acute, 2–6 weeks, and 6–12 weeks groups, respectively, recovered to an LSI of 90% or greater (*p* = 0.09, Cramer’s V = 0.27). All patients in the acute group were able to return to sports (*p* = 0.14; Cramer’s V = 0.28). **Conclusions**: Acute ACLR is advantageous for the early recovery of strength and performance without adverse events. Acute ACLR may shorten the time spent away from sports activities.

## 1. Introduction

Anterior cruciate ligament (ACL) injuries are serious traumatic injuries, with an estimated 350,000 cases occurring annually in the United States [1]. In Japan, approximately 3000 ACL injuries occur annually in junior and senior high school students [2]. ACL injuries are more common in physically active young people [3], and their number has increased over the past several decades [4,5,6].

ACL reconstruction (ACLR) is the recommended treatment for ACL injuries, especially for patients who wish to return to sports activities [7,8]. However, patients usually require a considerable amount of time to return to sports activities after ACLR because of graft remodeling. The remodeled ligament histologically differs from normal and has insufficient mechanical strength even 1 year after ACLR [9,10]. Furthermore, studies on ACL reinjury following ACLR have demonstrated that patients who return to sports activities earlier are at a higher risk of reinjury [11,12,13,14,15]. Recent reports have suggested that sports activities should be avoided for 2 years after ACLR [16]. Many medical institutions allow patients to return to sports activities 6–12 months after ACLR [11,17,18]; however, the period of inactivity is very long.

The inability to participate in sports activities happens not only postoperatively but also preoperatively. It has been reported that ACLR can cause joint contractures when inflammatory symptoms are present after an ACL injury [19,20,21,22,23]. Joint contracture is one of the factors that contributes to a decrease in sports activity level compared to that before the ACL injury [24]. Therefore, it is common practice to avoid ACLR in the acute phase to prevent joint contracture [25]. However, delayed surgical treatment can increase the incidence of meniscal tears and cartilage lesions [26,27,28,29,30,31] and the time required to return to sports. Additionally, even short periods of detraining can have adverse effects on physical function, including changes in body composition and reduced muscle cross-sectional area, maximal oxygen consumption, and performance [32,33,34].

Currently, minimally invasive arthroscopic ACLR is used instead of open surgery [21,22,25]. Rehabilitation protocols have also advanced, including shorter postoperative immobilization periods [22]. Recent reports have shown that acute ACLR and rehabilitation protocols do not cause joint contractures [35,36,37]. This, in addition to patient demand for an early return to sports, has led some medical institutions to perform acute ACLR. However, meta-analyses have not recommended acute ACLR [38,39]. This is because the aforementioned reports on acute ACLR are not different from conventional reconstruction in terms of joint range of motion, knee joint stability, and patient-oriented outcomes, and do not demonstrate the advantage of acute ACLR [35,36,37].

This study aimed to determine the effects of acute ACLR on postoperative outcomes, including joint stiffness, muscle strength, performance, and return to sports. The hypothesis was that there would be no difference in the knee range of motion or stability between acute ACLR and conventional reconstruction and that muscle strength, performance, and return to sports would be better with acute ACLR.

## 2. Materials and Methods

**Figure 1:**
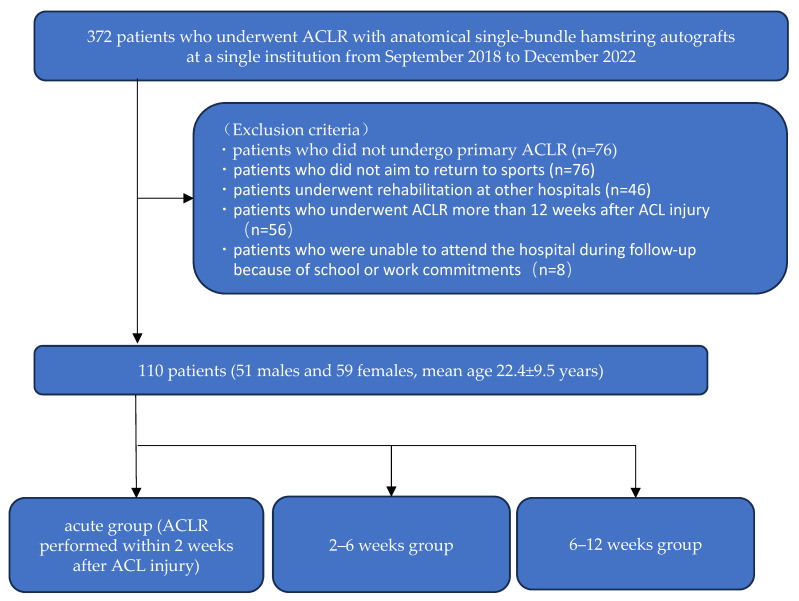
Flowchart of patients. ACL: anterior cruciate ligament; ACLR: ACL reconstruction.

This study was approved by the Ethics Committees of Ichihara Hospital (approval no. 1901) and Ibaraki Prefectural University of Health Sciences (approval no. e327). The approval waived the need for written informed consent due to the retrospective nature of the study. However, we maintained the opt-out policy mentioned on our hospital’s webpage, whereby eligible participants could withdraw from the study at any time. This study included 372 patients who underwent ACLR using anatomical single-bundle hamstring autografts at a single institution between September 2018 and December 2022. Patients who did not undergo primary ACLR (76 cases), those who did not aim to return to sports (76 cases), and those who underwent rehabilitation at other hospitals (46 cases) were excluded. Furthermore, we excluded 56 patients who underwent ACLR more than 12 weeks after ACL injury, as it has been reported that delayed surgical treatment increases the risk of associated injuries and results in poor postoperative outcomes [31,40,41,42]. Additionally, we excluded 8 patients who were unable to attend the hospital during follow-up because of school or work commitments. Finally, 110 patients (51 males and 59 females, mean age: 22.4 ± 9.5 years) were included in this study (Figure 1).

All 110 participants were divided into three groups based on the timing of ACLR following ACL injury: acute (ACLR performed within 2 weeks after ACL injury), 2–6 weeks (ACLR performed between 2 and 6 weeks after injury), and 6–12 weeks groups (ACLR performed between 6 and 12 weeks after injury) (Figure 1). The definition of acute ACLR is not standardized, and there is no clear definition [43]. Previous studies have commonly defined acute ACLR as ACLR within 2–3 weeks after ACL injury [19,37,39]. Therefore, in this study, we defined the acute ACLR group as ACLR within 2 weeks of ACL injury. Additionally, the control groups in previous studies were often defined as 6 weeks or later after ACL injury [19,36,37]; therefore, we used 6 weeks as the demarcation between the groups.

All patients underwent an arthroscopic, anatomical, single-bundle ACLR using a hamstring autograft. Bone tunnels were created inside the ACL attachment areas in the femur and tibia using the outside-in technique. The graft was fixed using endobuttons on the femoral side and screws on the tibial side. The surgical procedures were conducted by H.M., A.W., Y.Y., K.I., and T.K., each of whom had 28, 26, 14, 36, and 31 years of experience as a physician, respectively. The patients were hospitalized and rehabilitated for one week after surgery, and then continued rehabilitation one day a week until returning to sports. The postoperative rehabilitation protocol was as follows: immediately after ACLR, 1/2 partial weight bearing with a knee brace was allowed. Two-thirds of partial weight-bearing was permitted at 2 weeks, and full weight-bearing was permitted at 3 weeks after ACLR. Icing and non-steroidal anti-inflammatory drugs were initially administered to alleviate postoperative pain and swelling. Knee joint extension range of motion exercises were initiated on the day after surgery and knee joint flexion range of motion exercises were initiated 1 week postoperatively. Patients with meniscal sutures began knee flexion exercises 2 weeks postoperatively. The isokinetic strengths of the quadriceps and hamstrings were assessed at 60°/s using the Biodex System 3 (Biodex Inc., Shirley, NY, USA) 3 months postoperatively, during which jogging was permitted. Jumping exercises were started 5 months postoperatively, and specialized sports exercises were started 6 months postoperatively. Recommendations for return to sports were made based on the test results of isokinetic strength and single-leg hop (≥90% of healthy contralateral limb) and agility footwork (favorable knee alignment, especially no valgus in cutting and jump landing). The physician conducted a comprehensive evaluation and approved the patients’ return to sports 9 months after ACLR.

The following factors were assessed at the time of surgery: age, sex, height, weight, body mass index (BMI), activity level (competitive or recreational sports), sports events, and presence of meniscal tears. This study investigated several factors in each patient, including knee joint range of motion, knee joint stability (measured using the anterior drawer, Lachman, and pivot shift tests), patellofemoral joint pain, performance tests (single-leg hop) at 6 months postoperatively, isokinetic knee strength at 3 and 6 months postoperatively, return to sports, number of joint manipulations, and number of graft ruptures. After ACLR, the patients were monitored for 15 months, and we compared these parameters between the three groups. Adverse effects of detraining have been observed in highly trained athletes [33]. Graft rupture is more common in competitive sports than in recreational sports [11,43]. Therefore, we conducted a similar study in competitive-level athletes (*n* = 67). We defined competitive-level athletes as those who participate in sports at least 4 days per week and engage in competitive events.

The physician evaluated the knee joint stability using three tests (anterior drawer, Lachman, and pivot shift tests). A positive result in any test indicates instability, whereas a negative result in all three tests indicates stability. The Biodex System 3 (Biodex Inc.) was used to assess the isokinetic strength of the quadriceps and hamstrings at 60°/s. We extracted the peak torque value of three repetitions and calculated the ratio between the involved and uninvolved limbs as the Limb Symmetry Index (LSI). The single-leg hop test involves hopping forward as far as possible on one leg, with the great toe of the testing leg on the start line. The hopped distance was measured at the rear of the foot upon landing. Both limbs were tested, and no restrictions were imposed on the participants regarding the use of arm movements. The patients performed two trials following three practice sessions and were required to maintain the landing position for a minimum of 2 s. Unsuccessful hops were classified as loss of balance, extra hop on landing, or touching of the contralateral lower extremity. The maximum distance was used to calculate the LSI. Returning to sports was defined as the point at which the patient could resume practicing without restrictions. In cases in which an ACL graft rupture was suspected, orthopedic physicians specializing in sports injuries conducted physical evaluations. All ACL graft ruptures were confirmed by magnetic resonance imaging or arthroscopy.

All statistical analyses were performed with R Commander 4.3.2 (CRAN, freeware) [44]. To compare sex, activity level, presence of meniscal tears, sports events, knee joint stability, presence of patellofemoral joint pain, single-leg hop test, return to sports, and number of graft ruptures, either the Pearson’s chi-square test or Fisher’s exact test was used. After performing Shapiro-Wilk tests to determine the normality of continuous variables such as age, height, weight, BMI, time from ACL injury to ACLR, range of motion, and isokinetic strength, we applied either a one-way ANOVA or Kruskal-Wallis test. When significant differences were found, we used multiple comparison procedures, such as the Tukey and Steel-Dwass tests. Statistical significance was set at *p* < 0.05, and effect sizes were calculated for each parameter.

## 3. Results

### 3.1. Result 1 (All Patients)

**Figure 2:**
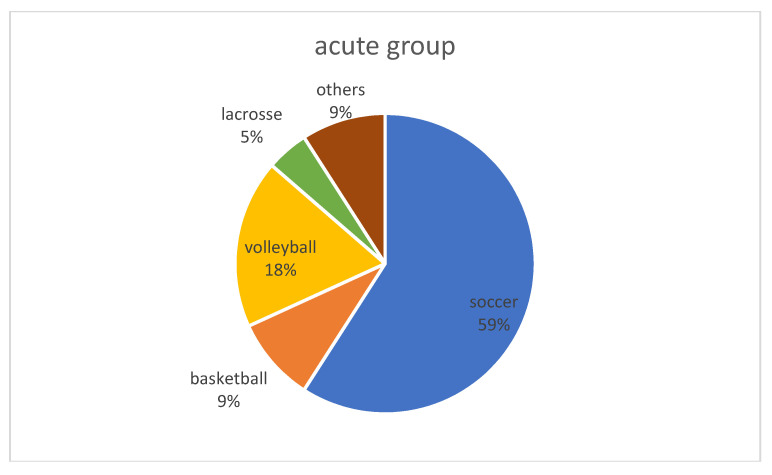

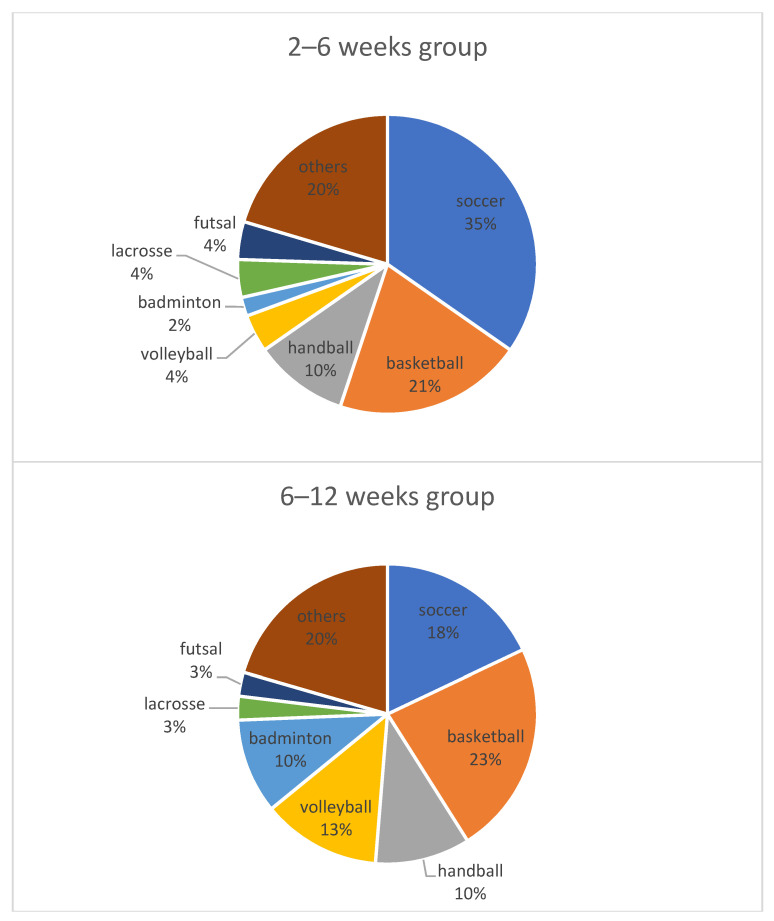
Sports events for all patients in each group. No significant difference was found between the groups (*p* = 0.10).

The study included 22, 49, and 39 patients in the acute, 2–6 weeks, and 6–12 weeks groups, respectively. Sex, age, height, weight, BMI, presence of meniscal tears, and sports events were similar between the three groups (Table 1) (Figure 2). Regarding activity level, patients in the acute group engaged in more competitive sports, while patients in the 6–12 weeks group participated in more recreational sports (*p* < 0.05).

Additionally, there were no significant differences in range of motion, knee joint stability, or patellofemoral joint pain between the three groups. However, only one patient in the acute group who underwent medial collateral ligament (MCL) repair experienced joint contracture and required joint manipulation 83 days after primary ACLR. Although some patients in the acute group had significant preoperative limitations of knee joint extension (−20°) or flexion (60°), they did not develop joint contracture postoperatively. Regarding isokinetic knee strength, the acute group exhibited significantly greater quadriceps strength at 3 months postoperatively than the other groups (acute group vs. 2–6 weeks group, *p* < 0.05; acute group vs. 6–12 weeks group, *p* < 0.05). No significant differences were observed in the other parameters (Table 2).

### 3.2. Result 2 (Competitive Sports Level Cases Only)

**Figure 3:**
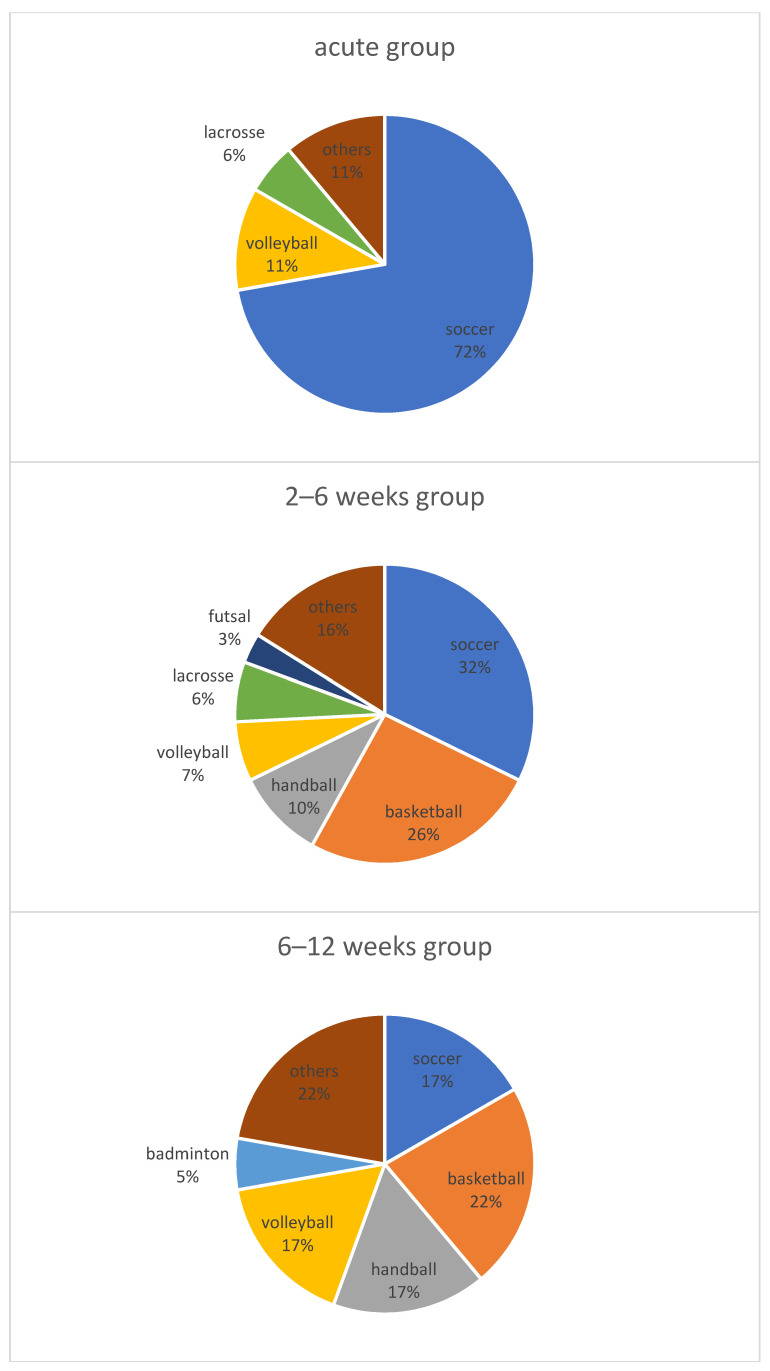
Sports events for each group in competitive-level athletes only. The proportion of sports events was significantly higher for soccer and lower for basketball in the acute group, but lower for soccer in the 6–12 weeks group (*p* < 0.05).

This study included 18, 31, and 18 athletes at a competitive level in the acute, 2–6 weeks, and 6–12 weeks groups, respectively. There were no significant differences between the three groups in terms of sex, height, weight, BMI, or the presence of meniscal tears. However, age was significantly higher in the acute group than in the 6–12 weeks group (*p* < 0.05), and the acute group had significantly more soccer and less basketball, while the 6–12 weeks group had less soccer (*p* < 0.05) (Table 3) (Figure 3).

There were no significant differences in range of motion, knee joint stability, or patellofemoral joint pain between the three groups. However, the isokinetic strength of the quadriceps at 3 months postoperatively was significantly higher in the acute group than those in the other groups (acute group vs. 2–6 weeks group, *p* < 0.01; acute group vs. 6–12 weeks group, *p* < 0.01). There was no significant difference in the isokinetic strength of the quadriceps at 6 months postoperatively between the three groups (*p* = 0.11, η^2^ = 0.07) (Table 4). Regarding the single-leg hop test at 6 months postoperatively, a statistically significant number of patients in the acute group were able to perform the test (*p* < 0.05, Cramer’s V = 0.31). The single-leg hop test showed that 66.7%, 38.7%, and 33.3% of the patients in the acute, 2–6 weeks, and 6–12 weeks groups, respectively, recovered to an LSI of 90% or greater (*p* = 0.09, Cramer’s V = 0.27). All patients in the acute group were able to return to sports (*p* = 0.14; Cramer’s V = 0.28). Graft rupture occurred in one patient in the acute group (5.6%), three patients in the 2–6 weeks group (12.9%), and three patients in the 6–12 weeks group (16.7%). One patient in the 2–6 weeks group and two patients in the 6–12 weeks group returned to sports at their own discretion and suffered a reinjury.

## 4. Discussion

The objective of this study was to compare the postoperative range of motion and knee joint stability between acute and conventional ACLR. Additionally, we evaluated the effects of acute ACLR on muscle strength, performance, and return to sports activities. The acute ACLR was defined as ACLR within two weeks of the ACL injury, while the conventional ACLR was defined as ACLR occurring six weeks or later after the ACL injury. Our findings indicated no significant differences in the postoperative range of motion or knee joint stability between acute and conventional ACLR. The results showed the superiority of acute ACLR in terms of muscle strength, performance, and return to sports, although there was a case that led to joint manipulation.

No significant differences in the range of motion were observed between the three groups. Even patients with a markedly limited preoperative range of motion did not develop postoperative joint contractures. This was consistent with previous studies [35,36,37] and suggests that joint contracture is unlikely to occur with acute ACLR using current surgical techniques and rehabilitation protocols. However, only one patient with an MCL injury, who underwent ACLR 13 days after the ACL injury, required postoperative joint manipulation. Petersen et al. reported that acute ACLR in patients with MCL injury complications often led to joint contracture [45]. Our results also suggest that acute ACLR should be avoided in patients with concomitant MCL injuries.

Regarding postoperative muscle strength of the quadriceps, the acute group exhibited significantly higher strength at 3 months and a trend towards higher strength at 6 months (no significant difference, effect size, medium). Previous studies have reported that acute ACLR often results in postoperative muscle weakness [21,25]; however, this was not observed in the current surgery. In contrast, a higher trend was observed. Studies have shown that a longer waiting period for surgery in patients with ACL injuries can result in preoperative muscle weakness, which, in turn, can lead to postoperative muscle weakness [46,47]. Acute ACLR performed before the onset of preoperative muscle weakness may result in higher postoperative muscle strength.

In the performance testing at 6 months postoperatively, more patients in the acute group were able to perform the test (*p* < 0.05) and recovered to 90% or more of the LSI (effect size, small). Eriksson et al. reported the isokinetic knee strength and single-leg hop performance at 6 months postoperatively of patients who had undergone ACLR within 8 days of ACL injury and between 6 and 10 weeks after ACL injury [48]. The study found that 12.1% of patients had an LSI of 90% or greater in knee extensor strength within 8 days of the ACL injury, and 5.9% of patients had an LSI of 90% or greater between 6 and 10 weeks after the injury. Additionally, 47% of patients had an LSI of 90% or greater in single-leg hop within 8 days of the ACL injury, and 21% of patients had an LSI of 90% or greater between 6 and 10 weeks after the injury. These results suggest that acute ACLR is beneficial, and our findings are consistent with these results. Lower extremity muscle strength is a crucial factor affecting performance [49,50]. Inadequate muscle strength can lead to poor alignment and fear of landing [13,51], which can make it difficult to undergo a performance test. The acute group, which had faster muscle recovery, progressed according to the postoperative rehabilitation protocol and may have had no difficulty in undergoing performance testing. Additionally, all patients in the acute group were able to return to sports (effect size, small). The high rate of return to sports following acute ACLR may be attributed to the rapid recovery of muscle strength and performance, which may be considered an advantage of acute ACLR.

Graft ruptures were observed in one (5.6%), four (12.9%), and three patients (16.7%) in the acute, 2–6 weeks, and 6–12 weeks groups, respectively. Graft rupture commonly occurs within the first year of surgery [12,13]. Additionally, there have been reports of graft ruptures after returning to sports without medical clearance [52]. Although a prolonged preoperative waiting period may result in a longer absence from sports and impatience in returning to sports, previous reports have not investigated the relationship between the preoperative waiting period and graft rupture. Therefore, further investigation of the relationship between the timing of ACLR and graft rupture is necessary to increase the number of cases in the future.

Reportedly, patellofemoral joint pain is more common in patients with poor muscle strength after ACLR [53,54]. Wang et al. found that patellar cartilage injuries were more common in patients with knee extensor strength with LSI less than 80% after ACLR [54]. In this study, we found no significant difference in patellofemoral joint pain between the groups at 6 months postoperatively. This was likely because the LSI of knee extensor strength averaged 80% in all groups, indicating a good recovery. Therefore, we concluded that the timing of ACLR does not affect the patellofemoral joint pain in patients within 3 months of ACL injury.

This study had several limitations. First, the sample size of the study was small. Although previous reports have used several methods to verify the results by comparing ACLR groups within 3 weeks and after 6 weeks following ACL injury [37,38,39,48], there are currently many cases in which ACLR is performed between the two groups. Therefore, this study used multiple comparisons for three-group comparisons. This study included all patients who underwent ACLR at a single institution using the same protocol over the past 4 years. However, due to the coronavirus disease 2019 outbreak, several sporting events were canceled, which affected an increasing number of patients with ACLR who did not intend to return to sports. Consequently, the number of cases was insufficient for multiple comparisons. Given the small number of cases, the purpose of our study was validated by calculating both the *p*-value and the effect size. Second, because this was a retrospective study, the demographics of the participants were not identical between the three groups. It is not certain how the age of the participants or their sport discipline would have affected the results of this study. Multicenter studies should be conducted in the future to consider these limitations.

## 5. Conclusions

Except for patients with MCL injuries, acute ACLR is advantageous for the early recovery of strength and performance without adverse events, such as joint contracture or joint instability. Additionally, acute ACLR may shorten the time spent away from sports activities.

## Figures and Tables

**Table 1:** Patient characteristics.

	Acute Group	2–6 Weeks Group	6–12 Weeks Group	*p*-Value
Time from ACL injury to ACLR, day ^a^	7.8 ± 4.2 (1–14)	27.2 ± 7.9 (15–40)	58.7 ± 12.4 (43–82)	<0.01 *
Sex, *n*				
Male	10	24	17	0.88
Female	12	25	22	
Age, y ^a^	24.3 ± 10.2 (14–49)	21.9 ± 8.4 (12–55)	22.0 ± 10.5 (13–58)	0.20
Height, cm ^a^	164.8 ± 10.6 (149.0–183.7)	164.9 ± 9.2 (149.8–187.5)	163.2 ± 7.7 (149.2–178.8)	0.60
Weight, kg ^a^	62.5 ± 11.7 (44.2–85.0)	63.1 ± 13.2 (39.9–107.0)	61.9 ± 11.2 (45.4–88.2)	0.89
BMI ^a^	22.9 ± 3.0 (17.3–27.9)	23.0 ± 3.2 (17.9–34.9)	23.1 ± 3.1 (18.8–32.9)	0.99
Activity level, *n* (%)				
Competitive sports	18 (81.8%)	31 (63.3%)	18 (46.2%)	0.02 *
Recreational sports	4 (18.2%)	18 (36.7%)	21 (53.8%)	
Meniscal tears, *n* (%)				
No	7 (31.8%)	22 (44.9%)	18 (46.2%)	0.50
Yes	15 (68.2%)	27 (55.1%)	21 (53.8%)	
MCL injury, *n*	1 (MCL repair)	1 (MCL repair)	0	

^a^ Data are presented as mean ± SD (range: minimum–maximum). ACL: anterior cruciate ligament. ACLR: ACL reconstruction. BMI: body mass index. MCL: medial collateral ligament. * *p*-value < 0.05.

**Table 2:** Comparison of range of motion, knee joint stability, patellofemoral joint pain, and isokinetic strength test (all patients).

	Acute Group ^b^	2–6 Weeks Group	6–12 Weeks Group	*p*-Value
Knee joint extension range of motion ^a^	−0.2 ± 1.9 (−5.0–5.0)	−0.1 ± 1.2 (−5.0–5.0)	0.1 ± 1.1 (−3.0–5.0)	0.59
Knee joint flexion range of motion ^a^	142.9 ± 5.4 (130.0–155.0)	142.6 ± 5.0 (125.0–155.0)	142.1 ± 4.7 (135.0–150.0)	0.75
Knee joint stability test, *n* (%)				
Positive	1 (4.8%)	1 (2.0%)	0 (0%)	0.47
Negative	20 (95.2%)	48 (98.0%)	38 (100%)	
Patellofemoral joint pain, *n* (%)				
No	19 (90.5%)	41 (83.7%)	34 (89.5%)	0.70
Yes	2 (9.5%)	8 (16.3%)	4 (10.5%)	
Quadriceps strength (postoperative 3 m), LSI ^a^	73.4 ± 13.1 (41.1–93.0)	62.7 ± 14.1 (27.2–91.1)	63.3 ± 13.5 (35.7–109.8)	0.01 *
Hamstring strength (postoperative 3 m), LSI ^a^	74.0 ± 15.7 (41.5–102.1)	66.6 ± 13.6 (33.9–91.8)	71.4 ± 13.9 (47.0–108.7)	0.09
Quadriceps strength (postoperative 6 m), LSI ^a^	83.8 ± 12.5 (62.8–105.8)	76.7 ± 13.2 (39.0–102.4)	78.2 ± 13.6 (35.9–103.7)	0.12
Hamstring strength (postoperative 6 m), LSI ^a^	87.0 ± 9.5 (63.0–103.7)	87.2 ± 13.9 (52.6–127.6)	85.6 ± 12.6 (56.0–118.9)	0.83

^a^ Data are presented as mean ± SD (range: minimum–maximum). ^b^ One patient who underwent joint manipulation was excluded. * *p*-value < 0.05. LSI, Limb Symmetry Index ([Involved Limb ÷ Uninvolved Limb] × 100%).

**Table 3:** Patient characteristics (competitive-level athletes only).

	Acute Group	2–6 Weeks Group	6–12 Weeks Group	*p*-Value
Time from ACL injury to ACLR, day ^a^	7.0 ± 4.2 (1–14)	27.3 ± 8.4 (15–40)	54.6 ± 9.6 (43–75)	<0.01 *
Sex, *n*				
Male	8	13	6	0.77
Female	10	18	12	
Age, y ^a^	21.4 ± 8.0 (14–49)	18.0 ± 3.6 (12–31)	17.2 ± 6.4 (13–41)	<0.01 *
Height, cm ^a^	165.5 ± 11.1 (149.0–183.7)	164.4 ± 9.0 (149.8–187.5)	160.5 ± 8.3 (149.2–178.8)	0.25
Weight, kg ^a^	62.2 ± 12.1 (44.2–85.0)	63.5 ± 15.0 (39.9–107.0)	61.0 ± 11.5 (45.4–88.2)	0.81
BMI ^a^	22.6 ± 3.1 (17.3–27.9)	23.3 ± 3.7 (17.8–34.9)	23.5 ± 3.2 (20.4–32.9)	0.76
Meniscal tears, *n* (%)				
No	6 (33.3%)	13 (41.9%)	10 (55.6%)	0.40
Yes	12 (66.7%)	18 (58.1%)	8 (44.4%)	
MCL injury, *n*	0	0	0	

^a^ Data are presented as mean ± SD (range: minimum–maximum). ACL: anterior cruciate ligament. ACLR: ACL reconstruction. BMI: body mass index. MCL: medial collateral ligament. * *p*-value < 0.05.

**Table 4:** Comparison of range of motion, knee joint stability, patellofemoral joint pain, isokinetic strength test, single-leg hop test, return to sports, and graft rupture (competitive-level athletes only).

	Acute Group	2–6 Weeks Group	6–12 Weeks Group	*p*-Value
Knee joint extension range of motion ^a^	−0.3 ± 2.1 (−5.0–5.0)	0.0 ± 1.3 (−5.0–5.0)	0.1 ± 1.5 (−3.0–5.0)	0.81
Knee joint flexion range of motion ^a^	143.3 ± 5.4 (130.0–155.0)	143.2 ± 4.6 (135.0–155.0)	140.3 ± 3.7 (135.0–145.0)	0.08
Knee joint stability test, *n* (%)				
Positive	1 (5.6%)	1 (3.2%)	0 (0%)	1.00
Negative	17 (94.4%)	30 (96.8%)	17 (100%)	
Patellofemoral joint pain, *n* (%)				
No	16 (88.9%)	26 (83.9%)	15 (88.2%)	1.00
Yes	2 (11.1%)	5 (16.1%)	2 (11.8%)	
Quadriceps strength (postoperative 3 m), LSI ^a^	76.4 ± 9.6 (63.1–93.0)	63.8 ± 11.9 (29.1–81.1)	63.4 ± 16.2 (35.7–109.8)	<0.01 *
Hamstring strength (postoperative 3 m), LSI ^a^	72.1 ± 15.5 (41.5–97.0)	65.3 ± 11.9 (37.4–89.8)	74.7 ± 16.0 (47.3–108.7)	0.06
Quadriceps strength (postoperative 6 m), LSI ^a^	86.6 ± 11.2 (64.5–105.8)	80.3 ± 10.4 (39.0–93.7)	80.0 ± 15.8 (35.9–103.7)	0.11
90% LSI or greater, *n* (%)	7 (38.8%)	4 (12.9%)	4 (23.5%)	0.12
Hamstring strength (postoperative 6 m), LSI ^a^	86.5 ± 9.4 (63.0–97.8)	86.7 ± 13.2 (60.1–127.6)	87.9 ± 12.9 (64.5–118.9)	0.93
90% LSI or greater, *n* (%)	8 (44.4%)	11 (35.4%)	9 (52.9%)	0.59
Single leg hop test, *n* (%)				
Possible	17 (94.4%)	22 (71.0%)	10 (58.8%)	0.04 *
Impossible	1 (5.6%)	9 (29.0%)	7 (41.2%)	
90% LSI or greater, *n* (%)	12 (66.7%)	12 (38.7%)	6 (33.3%)	0.09
Return to sports, *n* (%)	63 (94.0%)			
Yes	18 (100%)	30 (96.8%)	15 (83.3%)	0.14
No	0 (0%)	1 (3.2%)	3 (16.7%)	
Graft rupture, *n* (%)	1 (5.6%)	4 (12.9%)	3 (16.7%)	0.57

^a^ Data are presented as mean ± SD (range: minimum–maximum). * *p*-value < 0.05. LSI, Limb Symmetry Index ([Involved Limb ÷ Uninvolved Limb] × 100%).

## Data Availability

The datasets generated and/or analyzed during the current study are available from the corresponding author upon reasonable request.
